# Four-week heat acclimation lowers carbohydrate oxidation of trained runners during submaximal exercise in the heat

**DOI:** 10.3389/fphys.2025.1581594

**Published:** 2025-07-17

**Authors:** Yixiao Xu, Chengjie Ye, Su Ma, Binghong Gao

**Affiliations:** ^1^ Human Phenome Institute, Fudan University, Shanghai, China; ^2^ School of Exercise and Health, Shanghai University of Sport, Shanghai, China; ^3^ School of Athletic Performance, Shanghai University of Sport, Shanghai, China; ^4^ Physical Education and Research Section, Shanghai Datong High School, Shanghai, China; ^5^ Faculty of Health Sciences and Sports, Macao Polytechnic University, Macao, Macao SAR, China

**Keywords:** heat acclimation, carbohydrate oxidation, trained runners, aerobic capacity, exercise

## Abstract

**Purpose:**

This study examined the effect of 4 weeks of heat acclimation (HA, 39°C ≤ target Tc < 40°C) on aerobic capacity in middle-and-long distance runners, with a focus on metabolic adaptation.

**Methods:**

Eighteen male middle- and long-distance runners were randomized into exercise group (C group, n = 9) or heat acclimation group (HA, n = 9). The runners in the C group performed regular exercise training in a thermoneutral environment (20°C < wet bulb globe temperature ≤25°C), whereas the runners in the HA group underwent four-week heat acclimation (39°C ≤ target coer temperature <40°C), 5 days a week, once a day, for a total of 20 sessions over 4 weeks.

**Results:**

After 4 weeks of interventions, the core temperature after incremental treadmill test in the HA group (38.2°C ± 0.1°C vs. 38.6°C ± 0.1°C, *p* = 0.045) was significantly lower than that in the C group. The 4-week HA decreased the 0.4°C core temperature. The VO_2_ (44.7 ± 1.6 vs. 43 ± 2.9 mL/min/kg, *p* = 0.008) and velocity (12.9 ± 0.7 vs. 12.4 ± 0.9 km/h, *p* = 0.02) at the first ventilation threshold and the VO_2_ (55.9 ± 2.3 vs. 53.9 ± 3.1 mL/min/kg, *p* = 0.03) at second ventilation threshold increased compared with those in the C group. The carbohydrate oxidation (2.5 ± 0.1 vs. 3.1 ± 0.2 g/min, *p* = 0.01) at 75% V̇O_2_max and 85% V̇O_2_max exercise (3.4 ± 0.1 vs. 4 ± 0.2 g/min, *p* = 0.02) in the HA group decreased compared with that in the C group.

**Conclusion:**

Four-week heat acclimation reduced carbohydrate oxidation during submaximal exercise in the heat, indicating improved muscle glycogen utilization efficiency, which supports the enhancement of ventilatory thresholds and thermoregulatory adaptation, thereby improving aerobic capacity in the heat.

## Introduction

Around 70% of the global workforce (about 2.4 billion people) is now at risk of extreme heat (Wong, 2024). The Wet Bulb Globe Temperature (WBGT) is an international standard used to assess environmental heat load (Brimicombe et al., 2023). A WBGT of 33°C is recognized as a critical health threshold, at which only light workloads are recommended (Heo et al., 2019). Heat stress is known to impair 6%–16% of aerobic exercise performance in trained athletes (Casadio et al., 2017). Heat acclimation (HA), which refers to repeated exposures to heat environments over a set period, can enhance sweat and skin blood flow responses, fluid balance, cardiovascular stability, and thermal tolerance (Alkemade et al., 2021). These adaptations reduce thermal strain during exercise, typically seen as decreased core temperature and skin temperature, along with increased whole-body sweat rate and improved thermal comfort (Taylor, 2011). Investigating the optimal HA regiments is critical for responding to heat challenges. The optimal HA regiments require sufficient impulse balanced against detrimental overloading.

HA is classified as either short (≤7 days), medium (8–14 days) and long term (>15 days) in terms of training strategies (Xu et al., 2022a). Medium-term HA (1 or 2 weeks) is generally recommended to optimize competition performance (Racinais et al., 2015). Physiological adaptations, such as increased plasma volume and capacity for evaporative cooling, occur fast during medium-term HA, allowing for improved heat tolerance (Périard et al., 2016). However, increased erythropoiesis is not reported in HA studies with 10–14 days of exposure in trained athletes (Keiser et al., 2015; Karlsen et al., 2015). A series of recent study, where the intervention period was over 5 weeks, demonstrated that HA may translate into increased erythropoiesis (EPO) in trained athletes (Rønnestad et al., 2021; Lundby et al., 2023; Cubel et al., 2024). Besides, metabolic (i.e., decreased reliance upon carbohydrates as a fuel source) and thermoregulatory (i.e., reduced core temperature threshold for the onset of sweating and vasodilation) adaptations require at least 8 days to start (Chalmers et al., 2014), and 10–14 days of HA can reduce blood lactate during submaximal exercise in trained athletes (Lorenzo et al., 2010). Meanwhile, prior studies found that metabolic adaptations are still incomplete after 14 days (Patterson et al., 2004). Thus, the time course for HA studies to observe the metabolic adaptations should be long (>14 days). In mice, an increase in skeletal muscle citrate synthase activity and mitochondrial respiratory chain complex content were induced by 3 weeks of HA. Moreover, 4 weeks of HA can induce an enhancement in mitochondrial adaptation, as evidenced by PGC-1α and Pink1/Parkin upregulation (Tardo-Dino et al., 2021). Thus, long-term HA (4 weeks) ensures sufficient impulse for metabolic adaptation. Few studies notably reported the effects of 4-week HA on metabolic adaptation in trained athletes (Tyler et al., 2024). Metabolic adaptation is particularly important in endurance sports, in which glycogen depletion can cause fatigue (Xu et al., 2022b). Therefore, the effect of 4-week HA on metabolic adaptation in trained athletes need to be further investigated.

This study aimed to investigate the impact of 4-week HA on aerobic capacity, with a special focus on metabolic adaptations. This study hypothesized that 4-week HA can improve the metabolic adaptation in trained athletes, providing insights into the metabolic mechanisms that underline aerobic capacity in the heat and help athletes or workers exposed to heat to understand how long-term HA improves aerobic capacity.

## Methods

### Participants

Eighteen male middle-and-long distance trained runners who underwent HA in the latest 3 months were included. The characteristics of the runners are presented in Table 1. All participants signed informed consent forms. This study was conducted in accordance with the Helsinki Declaration and approved by the Ethics Committee for Scientific Research at Shanghai University of Sport (approval number: 102772022RT099).

**TABLE 1:** Characteristics of runners (mean ± SEM).

Group	Age	Height (cm)	Weight (kg)	Training years	V̇O_2_max (mL/min/kg)
C	17.6 ± 0.3	172.8 ± 0.6	56.0 ± 1.2	3.6 ± 0.5	62.8 ± 0.6
HA	17.6 ± 0.4	175.4 ± 0.4	58.6 ± 0.8	3.9 ± 0.4	62.2 ± 0.6

### Study design

**FIGURE 1:**
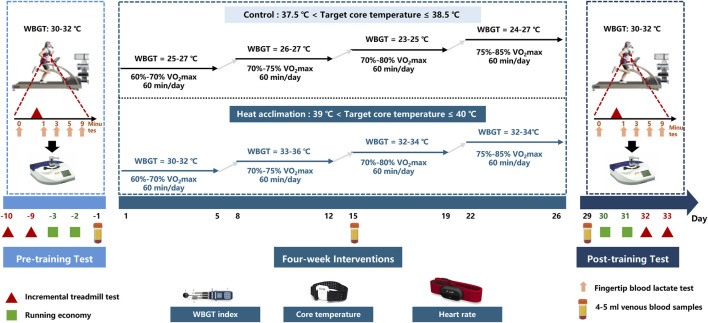
Study design of 4-week interventions. Before and after interventions, all runners completed the incremental treadmill test and running economy test on a separated days in the heat (30°C ≤ WBGT ≤ 32°C). During four-week interventions, The runners in the C group performed regular exercise training in a thermoneutral environment (20°C < wet bulb globe temperature ≤25°C), whereas the runners in the HA group underwent four-week heat acclimation (39°C ≤ target coer temperature <40°C), 5 days a week, once a day, for a total of 20 sessions over 4 weeks. The core temperature sensors and heart rate belt were worn during interventions. 4–5 mL venous blood samples were obtained from the antecubital vein on the baseline, second week and fourth week mornings at 6:00–7:00.

Runners were randomly assigned to the exercise group (C group, n = 9, minimum age of 16 years and a maximum age of 20 years) or the active HA group (n = 9, minimum age of 16 years and a maximum age of 22 years). The study designs are summarized in Figure 1. The C and HA groups received 4 weeks of interventions. Before and after the interventions, all participants completed the incremental treadmill test and running economy test on separated days in the heat (30°C ≤ WBGT ≤32°C). Ambient conditions were measured by a WBGT logger (HD32.2, Delta Ohm, Italy). The runners in the C group performed regular exercise training in a thermoneutral environment (20°C < wet bulb globe temperature ≤25°C), whereas the runners in the HA group underwent 4-week heat acclimation (39°C ≤ target coer temperature <40°C), 5 days a week, once a day, for a total of 20 sessions over 4 weeks. The core temperature sensors and heart rate belt were worn during interventions. Venous blood samples (4 or 5 mL) were obtained from the antecubital vein at baseline, second week, and fourth week between 6:00 a.m. and 7:00 a.m. (fasted for ≥8 h) for measurements. The serum was stored at −80°C for subsequent biochemical analysis after centrifugation (3,000 rpm, 15 min). The serum samples were used to analyze indicators such as plasma erythropoietin and testosterone. Plasma volume (PV) was calculated as follows: PV = (1 − hematocrit) × [a + b × weight (kg)], where hematocrit is expressed as a percentage, a = 1,530 (male)/846 (female), and b = 41 (male)/47.9 (female) (Ling et al., 2015; Dill and Costill, 1974).

### Interventions

The HA group completed a 4-week HA program consisting of daily exercise in the heat. The participants in the HA group were instructed to ran in the heat (30°C ≤ WBGT ≤ 36°C) at a work rate until reaching the target core temperature (39°C < target Tc ≤ 40°C) while wearing ore temperature sensors (CORE-TeamBundle, greenTEG AG, Switzerland). The CORE wearable sensor (4 cm × 5 cm × 0.8 cm) estimates Tc on the basis of the measurements of skin temperature, heat flux, and heart rate. Thereafter, external exercise intensity was adjusted as appropriate to maintain the target core temperature for a total session duration of 60 min.

The participants in the C group ran in a thermoneutral environment (23°C ≤ WBGT ≤ 27°C, 37.5°C < target Tc ≤ 38.5°C) while wearing core temperature sensors (CORE-TeamBundle, greenTEG AG, Switzerland). They were instructed to adjust their work rate to elicit the similar sRPE as reported by the participants in the HA group on the equivalent intervention day. The rating of perceived exertion (RPE) was measured 10–15 min after exercise, utilizing an adapted version of the Borg CR-10 scale. The CR-10 scale is a 11-point Likert scale, varying from 0 to 10 (Foster et al., 2001; Foster et al., 1996). The session-RPE (sRPE) method considers the intensity and duration to calculate the training load as follows: sRPE = RPE × session duration (min). The weekly sRPE is the sum of sRPE within the week: each week = 5 days × 60 min × RPE score (0–10).

### Incremental treadmill test in the heat

Incremental treadmill test was used to access runners’ aerobic capacity by using Cortex Metalyzer 3B (MetaLyzer 3B, CORTEX, Germany) on a motorized treadmill (Saturn 300/100, h/p/Cosmos, Germany) in a temperate laboratory (WBGT: 30°C–32°C). The test started at 11 km/h, with an incremental increase of 1 km/h per min and each stage lasting 2 min, and incline was maintained at 1%. The participants were considered exhausted when any of the following three conditions occurred simultaneously: 1) V̇O_2_max plateaued, 2) respiratory exchange ratio exceeded 1.15, and 3) heart rate surpassed 90% of the predicted maximum heart rate. Core temperature was measured by the greenTEG CORE sensor, and heart rate was measured by a heart rate belt (Team Pro, Polar, Finland). Body mass was tested before and after the incremental treadmill test. First ventilatory threshold (VT1) identified via V-slope method (increase in V̇CO_2_ relative to V̇O_2_). Second ventilatory threshold (VT2) marked by a systematic rise in ventilation (VE/V̇O_2_ inflection). The formula for calculating sweat rate (SR) is as follows: SR (L·h^-^1) = (pre-exercise body weight − post-exercise body weight + fluid intake − urine output)/exercise time (Yeo et al., 2012). The blood lactate concentration (BLa) was determined from the fingertip’s capillary blood samples (C line Clinic, EKF, Germany) before and after the incremental treadmill test at 1, 3, 5, and 9 min. Lactate clearance (LC) was calculated using the following formula: LC = [(lactate initial − lactate delayed)/lactate initial] × 100/delay (expressed as %/min), where lactate initial represents the blood lactate concentration immediately after exercise, lactate delayed is the blood lactate concentration at post-5 and 9 min, and delay is the time interval of recovery delay (in min).

### Running economy in the heat

Running economy was used to access energy demand and utilization at 65% V̇O_2_max, 75% V̇O_2_max, and 85% V̇O_2_max running velocity for separate duration of 5 min by using Cortex Metalyzer 3B (MetaLyzer 3B, CORTEX, Germany) on a motorized treadmill (Saturn 300/100, h/p/Cosmos, Germany) in a temperate laboratory (WBGT: 30°C–32°C). Carbohydrate oxidation (CHO) (Jeukendrup and Wallis, 2005; Venables et al., 2005) and energy expenditure (EE) (Brychta et al., 2010) were calculated using the following formulas: CHO (g/min) = 4.59 × VCO_2_ (L/min) – 3.23 × VO_2_ (L/min) and EE (kcal/min) = 1.106 × VCO_2_ (L/min) + 3.941 × VO_2_ (L/min).

### Statistical analysis

Statistical analysis was conducted on SPSS (version 2, IBM Corporation) or GraphPad Prism (version 8.3.0). Two-way repeated measure ANOVA was conducted to evaluate the changes in the measured variables over time. Bonferroni-adjusted pairwise comparisons were used where appropriate to determine where differences occurred. *p* values <0.05 are denoted as *. Data represent the mean ± SEM.

## Results

### Environmental conditions and training load

No significant differences were found in the sRPE between the C and HA groups (*p* = 0.974, Table 2). The WBGT and Tc in the HA group were significantly higher than those in the C group during 4 weeks of interventions (*p* = 0.0007 and *p* < 0.0001, respectively; Table 2).

**TABLE 2:** sRPE, WBGT and Tc during 4 weeks of interventions (mean ± SEM).

Parameters	Groups	Week 1	Week2	Week 3	Week 4
sRPE	C	2,113 ± 205	2,250 ± 184	2,172 ± 189	1823 ± 170
	HA	2,139 ± 169	2,267 ± 175	2,211 ± 341	1867 ± 261
WBGT (°C)	C	25.5 ± 0.3	26.9 ± 0.2	24.7 ± 0.2	25.1 ± 0.6
	HA	30.9 ± 0.3^a^	35.7 ± 0.6^a^	31.1 ± 0.5^a^	33.0 ± 0.7^a^
Tc (°C)	C	38.1 ± 0.2	38.3 ± 0.2	38.1 ± 0.2	37.4 ± 0.1
	HA	39.5 ± 0.5^a^	39.7 ± 0.4^a^	39.6 ± 0.3^a^	40.4 ± 0.1^a^

^a^ Significant effect of HA, *p* < 0.05.

### Physiological adaptation

**FIGURE 2:**
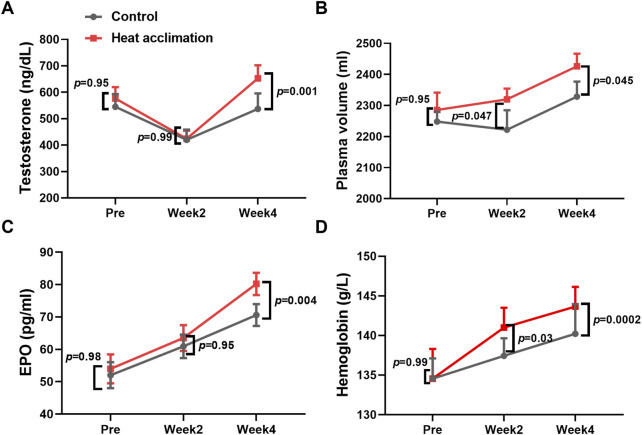
Results of testosterone, plasma volume, EPO, and hemoglobin during 4 weeks of interventions. **(A)** Testosterone during 4 weeks of interventions, **(B)** plasma volume during 4 weeks of interventions, **(C)** EPO during 4 weeks of interventions, and **(D)** hemoglobin during 4 weeks of interventions.

**FIGURE 3:**
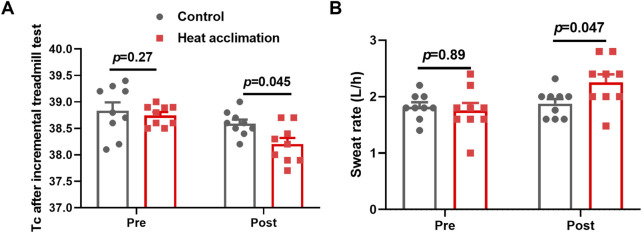
Core temperature and sweat rate of incremental treadmill test. **(A)** Core temperature of incremental treadmill test, and **(B)** sweat rate of incremental treadmill test.

Before the interventions, the core temperature, sweat rate, testosterone, plasma volume, EPO, and hemoglobin of the two groups did not significantly differ (p > 0.05, Figures 2, 3). After 2 weeks of interventions, testosterone (433.3 ± 36.6 vs. 419.8 ± 28.3 ng/dL, p = 0.99, Figure 2A) and EPO (60.9 ± 3.6 vs. 63.5 ± 4 pg/mL, p = 0.95, Figure 2B) still did not show significant differences between the two groups. The plasma volume (2,319.6 ± 34.7 vs. 2,222 ± 62.6 mL, p = 0.047, Figure 2C) and hemoglobin (141 ± 2.5 vs. 137.4 ± 2.1 g/L, p = 0.03, Figure 2D) in the HA group significantly increased compared with those in the C group. After 4 weeks of interventions, the Tc after incremental treadmill test in the HA group (38.2 ± 0. vs. 38.6°C ± 0.1°C, p = 0.045, Figure 3A) was significantly lower than that in the C group. Moreover, the 4-week HA decreased the 0.4°C core temperature. The sweat rate in the HA group (2.3 ± 0.1 vs. 1.9 ± 0.1 L/h, p = 0.047, Figure 3B) was significantly higher than that in the C group. The testosterone (653.1 ± 47 vs. 551 ± 53.9 ng/dL, p = 0.001, Figure 2C), plasma volume (2,425.9 ± 40.8 vs. 2,328.4 ± 48.6 mL, p = 0.001, Figure 2D), EPO (80.2 ± 3.4 vs. 70.6 ± 3.4 pg/mL, p = 0.004, Figure 2E), and hemoglobin (143.7 ± 2.5 vs. 140.2 ± 3.5 g/L, p = 0.0002, Figure 2F) in the HA group significantly increased compared with those in the C group.

### Incremental treadmill test in the heat

Before the interventions, the VO2 at VT1, VT2, and V̇O_2_max did not significantly differ (p > 0.05, Table 3). After 4 weeks of interventions, the VO2 (44.7 ± 1.6 vs. 43 ± 2.9 mL/min/kg, p = 0.01, Table 3) at VT1 and the VO2 (55.9 ± 2.3 vs. 53.9 ± 3.1 mL/min/kg, p = 0.031, Table 3) at VT2 in the HA group increased compared with those in the C group. Meanwhile, the V̇O2max showed no significant differences between the two groups (64.9 ± 1.6 vs. 67.1 ± 1.2 mL/min/kg, p = 0.08, Table 3). The velocity at VT1 in the HA group significantly increased compared with that in the C group (12.9 ± 0.7 vs. 12.4 ± 0.9 km/h, *p* = 0.02, Table 3).

**TABLE 3:** Results of incremental treadmill test before and after 4 weeks of interventions (mean ± SEM).

Parameters		Groups	Pre	Post	Interactive *p* value	Post *p* value
VO_2_ (mL/min/kg)	VT_1_	C	42.4 ± 1.2	43.0 ± 2.9	0.008	0.01
		HA	43.7 ± 0.9	44.7 ± 1.6^a^		
	VT_2_	C	51.0 ± 1.3	53.9 ± 3.1	0.035	0.031
		HA	51.0 ± 1.0	55.9 ± 2.3^a^		
	V̇O_2_max	C	62.8 ± 1.6	64.9 ± 1.6	0.18	0.08
		HA	62.2 ± 1.6	67.1 ± 1.2		
HR (bpm)	VT_1_	C	157.3 ± 2.9	155.1 ± 8.9	0.37	0.55
		HA	157.1 ± 2.8	158.2 ± 4.7		
	VT_2_	C	188.6 ± 1.4	186.9 ± 3.4	0.31	0.99
		HA	190.6 ± 1.2	186.7 ± 4.1		
	V̇O_2_max	C	190.9 ± 2.2	191.4 ± 5.5	0.28	0.89
		HA	188.9 ± 1.8	192.6 ± 5.2		
Velocity (km/h)	VT_1_	C	12.5 ± 0.4	12.4 ± 0.9	0.007	0.02
		HA	12.5 ± 0.3	12.9 ± 0.7^a^		
	VT_2_	C	17.3 ± 0.3	18.4 ± 0.7	0.32	0.13
		HA	17.5 ± 0.2	19.1 ± 0.5		
	V̇O_2_max	C	19.5 ± 0.2	20.1 ± 0.2	0.31	0.67
		HA	19.4 ± 0.2	20.3 ± 0.2		

^a^ Significant effect of HA, *p* < 0.05.

**FIGURE 4:**
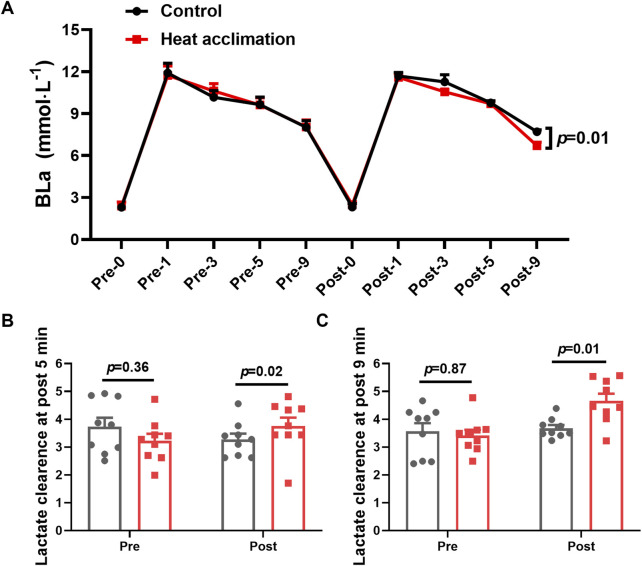
Results of blood lactate of incremental treadmill test in the heat. **(A)** Blood lactate of incremental treadmill test; **(B–C)** 5 min **(C)** and 9 min **(D)** blood lactate clearance of incremental treadmill test in the heat.

After 4 weeks of interventions, the HA group showed significantly lower BLa-9 (6.7 ± 0.7 vs. 7.8 ± 0.4 mmol/L, *p* = 0.01, Figure 4A) than the C group. The lactate clearance rate at 5 (3.8 ± 0.9 vs. 3.2 ± 0.8, *p* = 0.02, Figure 4B) and 9 min (4.7 ± 0.8 vs. 3.7 ± 0.3, *p* = 0.01, Figure 4C) in the HA group were higher than that in the C group after the 4-week intervention.

### Running economy in the heat

**FIGURE 5:**
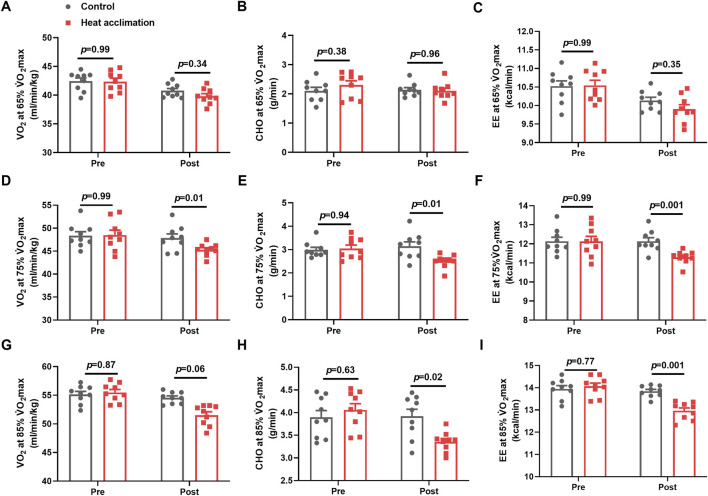
Results of running economy in the heat. Average oxygen uptake **(A)**, ratio of carbohydrates **(B)**, and energy expenditure **(C)** at 65%V̇O_2_max exercise intensity in the heat; average oxygen uptake **(D)**, ratio of carbohydrates **(E)**, and energy expenditure **(F)** at 75%V̇O_2_max exercise intensity in the heat; average oxygen uptake **(G)**, ratio of carbohydrates **(H)**, and energy expenditure **(I)** in 85%V̇O_2_max exercise intensity in the heat.

Before the interventions, the VO_2_, CHO, and EE at 65%, 75%, and 85% V̇O_2_max between the groups showed no significant differences (*p* > 0.05, Figure 5D). After 4 weeks of interventions, the VO_2_ (45.3 ± 0.5 vs. 48.4 ± 0.9 mL/min/kg, *p* = 0.01, Figure 5D), CHO (2.5 ± 0.1 vs. 3.1 ± 0.2 g/min, *p* = 0.01, Figure 5E), and EE (11.3 ± 0.1 vs. 12.2 ± 0.2 kcal/min, *p* = 0.001, Figure 5F) at 75% V̇O_2_max running velocity in the HA group decreased compared with those in the C group. The H group demonstrated a decrease in VO_2_ (51.5 ± 0.6 vs. 54.6 ± 0.3 mL/min/kg, *p* = 0.06, Figure 5G), CHO (3.4 ± 0.1 vs. 4 ± 0.2 g/min, *p* = 0.02, Figure 5H), and EE (13 ± 0.1 vs. 13.8 ± 0.1 kcal/min, *p* = 0.001, Figure 5I) at 85% V̇O_2_max running velocity compared with the C group.

## Discussion

This study evaluated the impact of 4-week active HA on aerobic capacity in the heat, with a focus on metabolic adaptation. The results showed that 4-week HA lowered the carbohydrate oxidation, indicating increased muscle glycogen utilization efficiency during submaximal exercise in the heat, thus supporting the thermoregulatory adaptation and improvement of aerobic capacity.

The 4-week HA reduced the 0.4°C core temperature during exercise in the heat, representing successful physiological adaptation. The decrease in core temperature during exercise induced by HA can reduce fatigue during exercise, as evidenced by the study showing that 4 weeks of HA increased the testosterone levels of trained runners. Testosterone is known to play a role in maintaining energy metabolism and muscle function (Crewther et al., 2018). It is associated with improved muscle recovery and reduced fatigue. In addition, a 21% increase in sweat rate, 4% increase in plasma volume, 2% increase in hemoglobin concentration, and 13% increase in erythropoietin observed after 4 weeks of HA contribute to thermoregulatory adaptation, allowing runners to dissipate heat more efficiently and enhancing oxygen transport. The increase in plasma volume during HA ranges from 3% to 37% (Patterson et al., 2014). Previous studies found that 23 male cyclists exercising for 60 min daily in a 37.8°C and 65% RH environment for 4 weeks had increased hemoglobin levels and cycling power output in the heat (Rønnestad et al., 2021). While the testosterone and erythropoietin levels did not increase at the end of second week in trained runners in the present study, a prior study did not observe erythropoietin adaptation following 2 weeks of HA (Karlsen et al., 2015). Thus, such thermoregulatory and cardiovascular adaptations induced by 4 weeks of HA are critical for aerobic capacity in trained runners.

Following the successful physiology adaptation induced by 4 weeks of HA, the runners performed the incremental treadmill test in the heat to access aerobic capacity. Cardiopulmonary exercise testing is a diagnostic method to evaluate aerobic capacity and cardiorespiratory function during exercise (Huggett et al., 2005). The results showed 4% increasement of VO_2_ at first ventilatory threshold and 3.7% increasement of VO_2_ at second ventilatory threshold after 4 weeks of HA, indicating that 4 weeks of HA improved submaximal exercise capacity in trained runners. Despite improvements in ventilatory threshold, the V̇O_2_max remained unchanged in the present study, likely due to the participants’ elite training status nearing their aerobic ceiling. Similarly, 12 male college students underwent 10 days of HA (50°C and 20% RH, target Tc at 38.5°C–39.0°C), resulting in increased ventilation thresholds during exercise (Beaudin et al., 2009). The improvement in ventilatory thresholds allows runners to perform at higher intensities without transiting to anaerobic metabolism and thus improving the submaximal aerobic capacity (Contreras-Briceño et al., 2024). Thermoregulatory and cardiovascular adaptations ensure an enhancement in oxygen transport to the muscle, delaying lactate accumulation and thus supporting the improvement of ventilatory thresholds (Lorenzo et al., 2011). The increased lactate clearance rate at 5 and 9 min of incremental treadmill test in the heat also support the improvement of ventilatory thresholds, which indicated the metabolic adaptations in response to the 4-week HA.

The runners performed the running economy test in the heat to further evaluate energy utilization and metabolic adaptation. The total carbohydrate oxidation decreased by 19% at 75% V̇O_2_max intensity and by 15% at 85% V̇O_2_max intensity after 4 weeks of HA. Prior studies found that the maximum rate of fat oxidation occurs at aerobic intensities of 60%–65% V̇O_2_max (Purdom et al., 2018). Carbohydrates are the primary fuel during moderate-to high-intensity aerobic exercise (65%–100% V̇O_2_max) because aerobic carbohydrate metabolism produces about 7% more ATP than fat (Kipp et al., 2018). During moderate-to high-intensity exercise, 15%–25% of the carbohydrate contribution comes from plasma glucose and 75%–85% comes from muscle glycogen, with over 80% of energy during high-intensity aerobic exercise (80%–100% V̇O_2_max) coming from muscle glycogen (Hargreaves and Spriet, 2020). Thus, lower carbohydrate oxidation at exercise intensities over 80% V̇O_2_max indicates decreased muscle glycogen utilization and increased muscle glycogen utilization efficiency (Areta and Hopkins, 2018). In the present study, the reduction in carbohydrate oxidation at 85% V̇O_2_max intensity demonstrated an improvement in muscle glycogen utilization efficiency after 4 weeks of HA. The decrease in carbohydrate oxidation observed after 4 weeks of HA represents a critical metabolic adaptation to support the enhancement of ventilatory thresholds. Enhanced muscle glycogen utilization efficiency ensures the less metabolic heat production and lower core temperature during exercise. The decreased carbohydrate oxidation induced by the 4-week HA becomes a crucial phenotype for endurance runners; it can not only optimize thermoregulation but also enhance aerobic capacity.

However, limitations should be considered in this study. First, the core temperature was monitored by the greenTEG CORE sensors continuously. While greenTEG sensors are effective in measuring surface temperature, accurately capturing core temperature, especially during high-intensity exercise, remains difficult. This difficulty may potentially affect the precision of interpreting physiological adaptation response to HA. Second, carbohydrate oxidation was calculated but not directly measured. Third, muscle glycogen utilization was not measured.

## Conclusion

In this study, the decrease in carbohydrate oxidation of trained runners during sub-maximum exercise in the heat induced by 4 weeks of heat acclimation indicates improved muscle glycogen utilization efficiency, supporting the enhancement of ventilatory thresholds and thermoregulatory adaptation, thereby improving aerobic capacity in the heat. The findings highlight that 4 weeks of heat acclimation as a strategy for athletes or workers to improve aerobic capacity and performance in the heat.

### What is already known on this topic

While the benefits of HA on aerobic capacity are well-documented, the optimal HA strategies remain unclear, and the reason why HA can improve aerobic capacity needs to be further investigated.

### What this study adds

The decrease in carbohydrate oxidation of trained runners during submaximal exercise in the heat induced by 4 weeks of HA indicates improved muscle glycogen utilization efficiency, which supports the enhancement of ventilatory thresholds and thermoregulatory adaptation, thereby improving aerobic capacity in the heat.

### How this study might affect research, practice, or policy

The study highlights the lower carbohydrate oxidation and paves the way for further research on muscle molecular mechanisms underlying HA. Four-week HA can be integrated into training programs for trained athletes or workers exposed to heat to optimize performance and safety.

## Data Availability

The raw data supporting the conclusions of this article will be made available by the authors, without undue reservation.
